# Transactions of the Medical Society of the County of Kings

**Published:** 1864-12

**Authors:** 


BUFFALO

### Medical and Surgical Journal.

VOL. IV. DECEMBER, 1864. No. 5.

### ART. 1.—*Transactions of the Medical Society of the County of Kings.*

REGULAR MEETING, OCTOBER, 1862.

*Tracheotomy in Diphtheria—Paper by* DR. WILLIAM GILFILLAN, *and remarks by* DR. JAMES M. MINOR.

The subject of the following paper is a case of diphtheria, in which the life of the patient was saved by tracheotomy.

Recoveries from this disease, after the performance of tracheotomy has been necessitated, are not frequent, and those which recover are, therefore, possessed of much interest.

In this case, the recovery is plainly and indisputably owing to the beneficial results of the operation, as the patient had not the advantages of pure air, careful nursing, nor even any great amount of cleanliness. Yet despite all these drawbacks, complete success has attended our efforts, and the patient is now entirely free from diphtheria, and able to walk about.

Without further preface, I shall briefly detail the circumstances of the case.

On the evening of October 2d, 1862, I was requested to visit the patient, John D., æt six. He had been healthy from birth, until two years ago, when he had a very severe attack of scarlet fever at Hartford, Conn. From that he completely recovered after a tedious convalescence.

Ten days before I was called to see him he began to complain of his throat. On the 27th of September he had great difficulty in swallowing.

On the 29th he was seen by a practitioner, (*irregular*, I believe,) who pronounced his disease "inflammation of the lungs," and gave him some powders which he said would move his bowels. They did not, however, produce any appreciable effect. He continued to get worse, and was again seen by this practitioner, but without any amelioration of his symptoms. His father desired to try other advice, and asked me to see him.

When I saw him, I found him sitting up in bed, gasping for breath. His respiration very hurried, stridulous and labored. All the auxiliary muscles of respiration were working violently. His face was covered with a heavy perspiration, his skin clammy and cool. At each inspiration, there was great sinking in at the epigastrium and the whole length of the sternum, as well as in a less degree at each side below the axilla.

On auscultation, very little air could be heard to enter the lungs; there were no râles. He could not speak, his pulse was feeble, slightly irregular, varying from 140 to 160 per minute.

There was a slight swelling of the neck at the angles of the jaw, the left cheek was slightly swollen likewise.

On examining the mouth and fauces, I found the tongue covered with a whitish fur; the velum palati presented a patchy appearance, pieces of membrane hanging in loose shreds from it. The tonsils, uvula and pharynx, as far as could be seen, were covered with membrane, and on the mucous membrane of the left cheek there was a spot the size of a quarter, covered with exudation.

The dyspnœa was so great that I considered the action of any medicine would be too slow to relieve the constriction of the glottis, before the brain and nervous centres would be too deeply prostrated by the circulation in them of blood very imperfectly aerated, as well as impregnated with the materies morbi of diphtheria.

To save the boy's life, whatever was to be done must be done quickly, or a few hours would end the struggle.

Fully convinced that tracheotomy alone afforded a chance for the boy's life, I explained the state of affairs to his parents. He was the only child remaining of seven, and they at once consented and requested me to operate as quickly as possible.

Accordingly, having secured the kind assistance of Prof. E. N. Chapman and Dr. W. Gardiner, I proceeded to operate at 9½ P. M. Chloroform was administered by Dr. Chapman, while Dr. Gardiner assisted in the operation. The trachea was opened a few rings below the cricoid cartilage.

No vessel of any size was seen or cut during the operation. (Our only light, I may remark, was four tallow candles.) There was not much blood lost, but some escaped into the trachea, and was ejected with difficulty. Considerable difficulty was experienced in introducing the tube after the trachea was opened. When it was introduced, the patient made little or no attempt to respire. Artificial respiration, by compressing the chest, etc., and the cold douche were employed, and after the lapse of fifteen or twenty minutes, he breathed easily and fully, and his pulse became fuller than before the operation.

I gave him now two or three tea-spoonsful of brandy. and ordered him a tea-spoonful of brandy every two hours with gr. ii. sulph. quinæ in solution. Soon he fell asleep and slept for several hours, occasionally waking up and coughing. I instructed his father to use a feather to remove the mucus collecting in the tube, and left for the night.

October 3d—morning. Breathing tranquil and free from pain, and no sinking in at the epigastrium. Pieces of membrane were separating from the fauces. Continue treatment, and give beef tea and eggs, ad libitum.

Evening. Considerable blueness of face, respiration hurried, and sinking in at the epigastrium as great as before the operation. This arose from the tube having become obstructed with hardened mucus, which required considerable force to remove it. I had introduced a double canula, and had cleaned the inner one thoroughly this morning, and yet in ten hours it had become nearly impervious. I now cleaned the inner tube again, and at once his respiration became perfectly tranquil. He brings up a good deal of tough mucus through the canula.

October 4th. Continues to improve; ate two eggs to-day and plenty of beef tea; swallows very slowly, but without much pain; pulse 110, of good strength, mind perfectly clear.

October 5th. Seems better, pulse good, 100 per minute, respiration easy, full expansion of chest. A little membrane still to be seen in the throat. The greater portion of the throat looks red and vascular. To continue brandy, and quinine and beef tea, and use an alum gargle. He brings up a great deal of tough glue-like mucus, which has a most peculiar mawkish odor; no appearance of membrane in it.

October 6th. Expression of countenance improved. Ate one egg, but took no medicine during the day. Pulse 100, tongue thickly furred. A little membrane to be seen on the back of pharynx. Urine has not been much, if at all diminished; to-day it was examined, but it contained no albumen. As I believe it is advisable, in all cases of tracheotomy for croup and diphtheria, to close the opening into the trachea as early as possible, lest by delay the larynx should undergo a chronic change, necessitating the permanent use of the tube, I took out the canula this afternoon at 5½ o'clock. I saw the patient at 9½ P. M., four hours afterwards. His breathing was labored and his face a little blue. He had great difficulty in expectorating the mucus. I again introduced the tube, and after which he slept most of the night.

October 7th. Not so well; pulse sinking, and 140 per minute; tongue thickly furred; continue medicine and gargle as before.

8th. In the evening felt better, pulse 100; good strength, no membrane to be seen in the throat, but it covers the wound to-day; cannot breathe through the larynx for any length of time without becoming blue in the face.

9th. Is quite lively to-day, pulse 100; is interested in hearing stories read to him. I applied a solution of nitrate of silver gr. xvi, aq. oz. j to the larynx, as I thought his inability to respire through it proceeded from œdema and fullness of the vocal cords, and not from any membraneous deposit which had probably separated before this time. The application caused the discharge of much glairy fluid from the throat.

10th. Much the same to-day; slept well last night; still continues to bring up tough mucus of a peculiar mawkish odor; applied the solution of nitrate of silver; I removed the tube and closed the orifice with lint; he breathed easily. Three hours afterwards the respiration was tranquil. In the evening I again applied the nitrate of silver. I removed the lint from the wound and brought the edges in contact with adhesive plaster eight days from the date of the operation.

11th. Patient slept very well last night, and feels well to-day. Breathing easy; swallowing is painful, and accomplished slowly, but without regurgitation; slight diarrhœa since yesterday; coughs considerably, and expectorates mucus streaked with blood. This morning he was able to say "good morning, sir," in a clear voice, when I entered the room. Continue treatment.

14th. Coughed very much last night, and slept badly; tongue very much furred; some isolated whitish patches are again visible on the soft palate and fauces; swallows without pain; breathes easily; pulse 120, feeble; takes but little nourishment; the wound in the throat is again covered with membrane. Omit quinine; take a tea-spoonful of brandy every four hours, and fifteen drops of ferri. tinct. mur., four times daily, beef tea, steak, eggs.

15th. Wound seems cleaner; less membrane on it; pulse 100, strong; tongue cleaning slowly; considerable cough, and a good deal of mucus ejected through the opening at times.

20th. Since last date he has improved wonderfully, and is walking about. He has still some cough; wound nearly closed, still some exuberant granulations; he has an excellent appetite, and sleeps well; expresses himself as quite well; his voice is still husky. In three or four days more, I expect the wound to be completely cicatrized; no air issues from it now.*

The foregoing case is, I think, a well marked example of a life saved by operative interference.

We see in it a case of diphtheria of moderate severity running its course, but unfortunately attacking the larynx, and producing very great constriction of the glottis, so that life was placed in most imminent peril. By opening the trachea, free admission was given to the air, the lungs once more expanded fully and freely. The blood which was loaded with carbonic acid gas and other impurities, was now thoroughly oxygenated and rendered capable of supplying life and vigor to all the organs and tissues of the body. It is true diphtheria still remained, weakening the organism by its septic influence; but time was given to combat it with appropriate remedies, and in the end the disease yielded to treatment steadily pursued. Such a happy result, unfortunately, does not attend all these cases; many die soon after operative interference.

Several causes combine to swell the mortality of such operations. These causes I shall discuss towards the end of this paper.

I would now draw attention to those symptoms which indicate the necessity of tracheotomy in diphtheria.

At the present time physicians, although differing in their pathological views of diphtheria, agree unanimously in their treatment of it. The supporting and stimulating plan of treatment is considered to be the most successful. In the details of treatment there are some minor differences, but the essential features are the same. The exudation on the throat or membrane, as it is generally called, is recognized as the local manifestation of a constitutional or general disease, just as the small pox pustule is a manifestation of small pox. Local applications are made to the throat, not

* The wound completely closed October 20th. The cicatrix is scarcely perceptible.

with the view of cutting short the disease, but of limiting the spread of the membrane, and favoring its dissolution. Frequently, when the disease is progressing well in other respects, the membrane extends into the larynx, as is shown by the suppressed husky voice, and after a time by difficulty of breathing, stridor, and finally by dyspnœa, more or less severe. This extension of the disease to the larynx is a severe complication, and often ends in death in a few hours, even when the patient's strength is pretty fair and the general symptoms not alarming.

In illustration of the rapidity with which death follows invasion of the larynx in this disease, I may cite two cases from my own practice. Last June I received a telegraphic message calling me to the village of Esopus, near Poughkeepsie, to see a child ten months old, which had been seized with diphtheria the previous day. I went prepared to perform tracheotomy, as the message intimated that the child's breathing was much affected. The child died about an hour before my arrival. The croupy breathing commenced at 10 A. M., and by 7½ P. M., the child was dead, retaining its strength almost to the last moment. The fauces were covered with false membranes.

The other case was a child three and a half years old, who had been suffering from sore throat for three days. There was some swelling of the neck. The fauces were covered thickly with false membrane. The pulse was 120 and feeble. The breathing was slightly croupy. The first visit was at 8 A. M. At midday there was great oppression of the respiration and sinking in at the epigastrium, on inspiration, some lividity of the face. The child would occasionally get up and walk round the room. I told the parents that the child would doubtless die unless tracheotomy were performed; that that alone gave any hope of recovery, but they declined the operation, as they "preferred to see the child die a natural death, rather than be butchered." He died in three hours afterwards. Although I expected a fatal result, I was surprised at the rapid termination of this case.

These cases of croupy diphtheria die much more quickly of suffocation than cases of real croup—*cynanche trachealis.* Nor is this to be wondered at, for the blood is both imperfectly aerated and loaded with the diphtheritic poison, which combination very quickly paralyzes the nervous system. Moreover, I believe diphtheritic exudation is poured out more rapidly than ordinary croupy exudation, and thus the rima glottidis is sooner closed to the ingress of air. This laryngeal complication of diphtheria seems more prevalent in some epidemics or some localities than others

Thus the cases at Tours, described by Bretonneau, who first clearly distinguished this disease, presented this complication more frequently than has been seen of later years, either in England or in this country. Indeed, so frequently did Bretonneau observe it that he believed that Dr. Home, of Edinburgh, who first wrote on cynanche trachealis, was mistaken in his views of that disease, and that it was really laryngeal diphtheritis: we know now that in this, Bretonneau erred.

We see, then, that *laryngeal* diphtheria runs a rapid course. Can it be. safely treated by the exhibition of medicines internally or locally?

In milder cases, various local applications may suffice, along with appropriate internal remedies. Yet generally the patient can bear no depressing remedy, as tart. antim; even the effort of vomiting may be too much for him in his enfeebled condition, and it may bring on an irritable state of the stomach, preventing the patient from retaining the food upon which his ultimate recovery depends. Even if, by vomiting, a portion of the membrane is thrown up, it is rapidly reproduced. Mercurials are not regarded with favor in diphtheria, and their action is too slow to contend with the rapid strides of this disease.

We have tracheotomy to fall back upon, but there is often an error committed in falling back on it *too late.*

*When the patient's strength is good, and the general symptoms fair, if there is great difficulty in respiration, as evidenced by slight lividity, stridor, and considerable sinking in of the parietes of the chest on inspiration, then I believe the operation of tracheotomy affords the best, if not the only hope of the patient's recovery, and is then strongly indicated.*

In this disease we cannot, with safety to the patient, defer tracheotomy to as late a stage as in croup, for reasons before mentioned. What then contra-indicates the performance of tracheotomy?

I would mention, as the only contra-indication, *too great exhaustion or depression* of the vital powers. It is impossible to set up an absolute standard upon this point; it must be left to the judgment and acumen of the practitioner.

Tracheetomy can only effect free admission of air into the lungs; if the patient is sinking from asthenia or from the toxic effects of the disease the, operation is useless or even injurious. There are other conditions which are frequently urged as contra-indicating the operation. These are:

I. Extreme youth of the patient.

II. The existence of pneumonia or bronchitis.

III. The extension of the membrane into the bronchi and lungs.

As regard the first, extreme youth, it is true that children under two years seldom recover after tracheotomy, yet there are cases recorded, of recovery under this age, some as young as six weeks. If the chance of a patient's recovery is small, that is no valid reason why we do not afford the patient that chance.

II The existence of pneumonia or bronchitis.

These complications undoubtedly lessen the recoveries materially, but neither of these diseases necessarily proves fatal. They make our prognosis more unfavorable, and they necessitate the supporting plan of treatment to be fully carried out. Pneumonia and bronchitis do not kill rapidly; they often prove fatal by asthenia; but laryngeal diphtheria, when it kills, kills quickly. The treatment of laryngeal diphtheria is, so to speak, limited to hours, the treatment of pneumonia and bronchitis extends to days. Why then should we hesitate to perform tracheotomy when it is indicated? Granted that the patient has pneumonia or bronchitis, let us relieve him of impending suffocation, and trust to after treatment to bring the pulmonary complications to a safe issue. Such, I venture to say, should be our rule of conduct in these complicated and fatal cases.

III. Let us now pass on to the third objection to the operation, viz: The extension of the disease to the bronchi and their divisions. This is undoubtedly a most important point. When the membrane extends into the minute bronchial ramificatious, the case is hopeless; when it exists in the larger bronchi, it may loosen and be expectorated, and does not necessarily cause death.

The practical question is, how do we recognize this state during life; what are its symptoms and physical signs?

Here, I fear, everything is vague and uncertain. The general symptoms are unsatisfactory data from which to form a diagnosis, and the physical signs are in the same category. When the dyspnœa is so great as to suggest the propriety of tracheotomy, auscultation is surrounded with difficulties, and is unreliable. The rapid breathing of the patient, and stridor from the larynx, mask or completely drown any other sign which is present. Besides, what acoustic phenomena would a thin uniform membrane, closely adhering to the lining membrane of the bronchi, produce? No râles whatever; it would merely diminish the amount of air passing through the bronchus; but when the larnyx is partly occluded, this effect is completely masked or merged in a greater.

If the membrane is partially detached in a large bronchus, it may give rise to a *reed-like* sound, but the same sound is produced occasionally by inspissated mucus.

There are, therefore, no symptoms, except the expectoration of bronchial casts—a very rare occurrence, which point conclusively to the extension of membraneous deposit into the bronchi, and their ramifications. We cannot be certain of its existence in any individual case, and the patient is entitled to the benefit of the doubt.

Let me cite one or two cases, to show that the impossibility of determining whether this membrane exists in the bronchi, or not, is real and practical, not imaginary or hypothetical. The following case is abridged from Dr. Greenhow's work on diphtheria:*

"Samuel, aged six years, a delicate scrofulous looking child, was seen for the first time on May 28th, 1858. He had just been brought home from the country, and had been ailing slightly for the previous four or five days. Tongue thickly covered with white fur; tonsils enlarged, and of a deep red color, without exudation; skin moist; pulse 90.

29, Thick exudation over tonsils and uvula, showing fibrinous structure under the microscope, and also a specimen of "oidium albicans;" the throat was brushed with equal quantities of dilute muriatic acid and water; to take four drops each of tinct. ferri. mur. and dil. muriatic acid every third hour; port wine and jelly.

30. Exudation less.

31. False membrane still covers the throat; breathing easy; patient does not seem depressed; continue treatment.

June 1st. A croupy cough came on in the night, and is well marked this morning; urine free from albumen; seen in consultation with Dr. Heslop; to have an acid gargle and a mustard emetic.

*Vespere.* The breathing is accompanied by marked stridor, which was easier after an application of the acid.

2d. Croupous symptoms very marked; a blister on the throat, an emetic of sulph. zinc and ipecacuanha.

*Vespere.* Countenance dusky; stridor great; voice suppressed; mustard emetic to be repeated; died at 4 A. M., June 3d.

P. M. examination. No pleuritic effusion; lungs emphysematous in front; collapsed posteriorly; deposit of tubercle in two bronchial glands,

---

* Page 109.

and also of the size of a horse bean in the right lung; a patch of false membrane at the bifurcation of the trachea; trachea reddened but free from exudation; larynx and epiglottis covered with lymph, as well also the pharynx, tonsils and uvula.

Here is a case where the membrane did not extend into the bronchi, yet the patient died of apnœa. No allusion is made to tracheotomy; yet from the post-mortem appearances, that operation would have given free ingress of air to the lungs, and after treatment might have carried the patient through. I incline the more to this opinion as it is expressly stated, May 31st, the day previous to the beginning of the croupy symptoms, "patient does not seem depressed."

The next case is from the Lancet.* It is a case where tracheotomy was performed, but the disease was found to extend to the remote bronchi. "Tracheotomy was performed upon a girl aged seventeen, by Mr. Prescott Hewett, a few days back, at St. George's Hospital. where she was apparently dying from diphtheria, having been admitted for that disease, und a the care of Dr. Bence Jones. When the canula was placed in the trachea, singular to relate, no air passed through it; it was therefore withdrawn and the finger introduced as far as the bifurcation. When the canula was reinserted the patient gave a slight cough and expectorated a distinct cylinder of croupy membrane, which was bifurcated, and possessed the form of the various ramifications of the larger bronchial tubes. This occurrence gave very marked relief; but the vital powers were already so enfeebled by the disease that she lived only a few hours after the operation. At a post mortem examination, the minutest ramifications of the bronchial tubes were found filled with lymph."

I shall conclude with the following case, likewise from the Lancet, †by Dr, Evans. "A boy aged ten years; diphtheria coming on very insidiously during nearly a month; treated by salines and the application of a solution of nitrate of silver to the throat; supervention of croupous symptoms treated by counter irritation, leeches, antimony, calomel, and chlorate of potash; asphyxia impending; tracheotomy and stimulating after treatment; death apparently from syncope, about twenty-six hours after the operation. After death a thick, false membrane, separable from the subjacent mucous membrane only with considerable force, was found to line the larynx and trachea, and to extend to the bifurcation of the latter; it

* Vol. II. 1859, page 64, American edition.

† Vol. II, 1859, p. 441, American edition.

probably, indeed, passed down into the lungs, but an examination of these organs was not permitted."

This patient underwent a considerable amount of treatment; salines, counter irritation, leeches, calomel, antimony, chlorate of potash, tracheotomy, and stimulation, but died syncopal. After reading such a case and its termination, I am compelled to ask, how might this case have progressed had counter irritation, leeches, antimony and calomel been omitted, and had tracheotomy been performed earlier? In other words, could this boy have recovered if his strength had been husbanded instead of squandered, and had tracheotomy been performed to relieve his dyspnœa, instead of applying leeches?

Dr. Greenhow's views on the subject of tracheotomy are expressed in the following paragraph:*

"Failing the success of other treatment in diphtheritic croup, the question will very properly arise whether, when dyspnœa is very urgent, the operation of tracheotomy should be performed. Upon this subject I have no personal experience, but the operation has, in this country, been almost always unsuccessful. On the other hand, I have had the opportunity, in two instances, of observing in post mortem examinations, that the false membrane extended a very short distance down the trachea, and in one of these, death appeared to have been caused by a partially separated membrane acting as an obstruction to the admission of air. Perhaps in this instance the performance of tracheotomy might have saved the patient, and when the case appears to be otherwise hopeless it would probably be right to give the patient the chance afforded by the operation, provided there should be no evidence of the extension of the disease to the bronchial tubes or of the existence of pneumonia, either of which would manifestly contra-indicate the performance of an operation, which must, under such circumstances, prove unavailing."

Dr. Greenhow's remarks on the non-success of this operation in diphtheritic croup, are, I believe, to a certain extent, borne out by experience. I find no successful case recorded in the Lancet since 1858, nor in the American Medical Times for two years past. What may be the explanation of this? Has the operation been considered so fatal as to be allowed to fall into disuse? I find that Bretonneau operated in twenty cases, with six recoveries; Trousseau, in one hundred and twelve cases, of which

* Op. cit., p. 159.

twenty-seven were successful, and Pancoast, in three, of which two recovered.

This operation is not to be judged by the same rules as other operations, many of which are, to a certain extent, "operations de complaisance." In diphtheria this operation is not thought of until life is placed in imminent peril, and those who recover are snatched from the jaws of death, Everything is to be gained by the operation, and nothing lost. Dr. Greenhow thinks that the extension of the disease to the bronchial tubes, or the existence of pneumonia, contra-indicate the operation. I believe I have shown that it is usually impossible to decide whether the bronchi are affected or not. His objection to operating where pneumonia exists is founded on theoretical considerations, for children affected with cynanche trachealis and pneumonia have recovered after tracheotomy, and why not with diphtheritic croup and pneumonia? Having now discussed the indications for the performance, operation, and the objections that are urged against it, and having shown that it promises a fair success in cases otherwise hopeless, I would draw attention to the causes which produce the high rate of mortality attending this operation. These causes may be conveniently analyzed in three groups:

I. The causes acting prior to the operation.

II. Those which are incident to the operation itself.

III. Those which arise subsequently to the operation.

And first, *those which act prior to the operation.* In this division may be classed a weak constitution and a protracted illness, neither of which, unfortunately, can the physician control.

The employment of depressing remedies in the early stages of the disease is a fruitful source of death, and is to be deprecated accordingly. Calomel, antimony and ipecacuanha should certainly be avoided, under almost any circumstances. Sulphate of zinc, mustard or alum, will produce vomiting, if that is thought advisable, and they have the decided advantage of antimony and ipecacuanha, in not producing depression or diarrhœa.

Another source of death is the postponement of the operation until the patient is in extremis, when, after hours of dyspnœa, the final struggle is at hand. To operate in such circumstances is but a forlorn hope. Let us operate, therefore, if practicable, when the patient's strength is as little reduced as possible.

II. The causes of death incident to the operation itself,

Hemorrhage, during the operation, may cause death, or greatly weaken the already depressed vital powers. This is to be guarded against by carefully looking out for large veins, and by cutting directly in the middle line, where, in the normal distribution, no vessels of consequence are met. Hemorrhage into the trachea may cause obstruction of the respiration, or even suffocation. To obviate this, the best plan is to insert the canula as quickly as possible after the trachea is opened. This is a nice point, and is only to be acquired by practice. When once inserted, the pressure of the tube staunches the flow of flood, and at the same time the circulation through the heart is facilitated by the free admission of air to the lungs, and the congestion of the venous system is thereby relieved. It is a matter of vital importance that the tube inserted, and through which the patient respires, should be as large as possible. Trousseau first drew attention to this point, that many persons died after tracheotomy from the tube introduced being too small to permit air enough to enter the lungs for the oxygenation of the blood. This defeats the very object of the objection, and renders it nugatory.

The canula should be as large as the trachea will admit of being introduced, and in children it should be double, to give facility for cleansing the tube, and removing the mucus which collects in it and proportionately narrows its calibre. It is essential also to have an attendant with the patient, who is able to keep the tube clear.

III. Those causes which arise subsequently to the operation.

These are varied, some springing from the operation, and some accidental. Diarrhœa occasionally supervenes and carries off the patient. More frequently the lungs are affected by the direct admission of cold air, and pneumonia and bronchitis follow.

After every case of tracheotomy there is, I think, more or less bronchitis where the patient survives the first few hours, but it gradually disappears. A more serious form of the disease may occur, or pneumonia may arise. In such circumstances, depletion in any form is admissible, and we must rely solely on the supporting treatment, beef tea, eggs and milk, with stimulants, and these must be given freely, judged not by the quantity but by the effect. The membraneous exudation may extend to the small bronchial tubes, in which case our efforts will be of little use, still the supporting plan is indicated.

When the patient escapes these dangers, and shows evidence of recovery, our next solicitude should be the removal of the tube, and the restoration of the respiration through the larynx as early as possible. If this is not done soon, the larynx, from disuse, contracts, so as to be too small for respiration. We have examples of the constriction of other canals where fistulous openings exist in them; thus the urethra diminishes in size in perineal fistula, and the intestinal canal shrinks below the opening in artificial anus. It is a grievious affliction to be condemned to breathe through a acnula for the term of one's natural life. When tracheotomy is performed for croup or diphtheritic laryngitis, we should try to withdraw the tube sometime between the fifth and fourteenth day. The conditions which demand the operation in these cases are transitory, and the sooner the canula is withdrawn the better, both as regards the recovery of voice and escape from pulmonary complications. On withdrawing the canula, the patient must be carefully watched for some hours, and if there is any difficulty of respiration, it should be quickly reinserted. In the case read before you, I removed the tube finally eight days after the operation, and the next day the boy could speak easily.

I have thus attempted, imperfectly, I am well aware, to discuss the propriety of tracheotomy in diphtheritic laryngitis; the indications for its performance, and the causes which give the operation its high mortality, in the hope that its discussion in this society may bring the experience of many to bear on this very important subject, and may perhaps cause the more general adoption of this operation, which *I am convinced will some timse save the lives of patients; more especially of children, when all other means fail.*

As the winter approaches, we may probably find the disease becoming more prevalent; at all events it is likely to be *endemic* for some time to come, so this discussion will be opportune.

Since the preceding paper was read I have operated in two cases of laryngeal diphtheria, both of which, however, proved fatal. The first case was in a child thirteen months old. The operation removed all difficulty in the respiration, but the patient died from asthenia, sixty hours after the operation.

The second case was a child five years old; the operation relieved the breathing, and strong hopes were entertained of the patient's recovery, but the difficulty of breathing returned, probably from the disease extending to the bronchial ramifications, and he died.

Subjoined is a short history of each case.

October 27th. I was asked by Dr. Hallett to see a patient that had been suffering from diphtheria for two days. The patient, a little girl only thirteen months old, was well developed for her age. and healthy, light hair and complexion, When Dr. Hallett first saw the patient, the diphtheritic exudation was well marked on the fauces. Dr. Hallett prescribed citrate of iron and quinine in full doses every three hours, and to have plenty of beef tea, etc, She progressed pretty well until last night, when the breathing became affected; for this several emetics of sulphate of zinc were given, but they failed to relieve the breathing, which gradualiy became worse. At 2½ P. M. the following was her condition: Face somewhat livid; surface covered with clammy perspiration; pulse very rapid and feeble; great gasping for breath; stridor and considerable sinking in at the epigastrium with each inspiration; fauces covered with false membrane. It was apparent she could not survive more than three or four hours, if as much. Tracheotomy was proposed as the only means which gave the least prospect of recovery, although from the tender age of the child there could not be as much hoped for from the operation as in an older person. After some hesitation the mother acceded to the operation; the father was absent from home.

I at once operated and introduced a good sized tube into the trachea. On account of the shortness of the neck and the great amount of adipose tissue, the operation was more difficult than ordinary, but as soon as the tube was introduced the breathing was instantaneously relieved, the bluish tinge left the face and she fell asleep. To continue the citrate of iron and quinine, beef tea and brandy,

October 28th. Seems easier; breathing easy; pulse 120; takes her nourishment well.

29th. Not so well to-day; lividity somewhat returning; pulse very feeble and compressible; cannot swallow; slight diarrhœa. She was ordered injections of beef tea and brandy, with a few drops of laudanum, to check the diarrhœa, every three hours.

*Vespere.* -Has retained the injections pretty well; had also an injection of gr. iii sulph. quinæ in solution; is more feeble; pulse very rapid and weak; no difficulty in respiration; very lttle mucus in the tube all day. She died calmly at 2 A. M., October 30th. No examination of body.

E. H., aged four years and ten months, of healthy parents. I was called to see this patient on the night of January 2d, 1863, in the middle of the night, and went prepared to perform tracheotomy, at the request of Dr. J. E. Clark. I found the patient breathing with some laryngeal noise, but still easily, face natural, no sinking in at epigastriem on inspiration.

Dr. Clark stated that he had been affected with diphtheria for six days previously. The treatment consisted of quinine and whisky; no local applications, I believe, had been made. The boy had done very well, was improving and sitting up, but on the 2d his mother permitted him to roam about the house, as he seemed quite well. At seven P. M. his breathing became croupy; at ten o'clock Dr. Clark was called, and found the dyspnœa very severe; the dyspnœa increased rapidly, and Dr. Clark thought tracheotomy would be required.

In the interval between sending the messenger and my arrival, the paroxysm yielded, and when I arrived he was much easier. The operation was not considered necessary then. The quinine and whisky were ordered to be continued freely.

On the 3d the dyspnœa steadily increased; there came on great lividity of the face, great stridor and sinking in of the parietes of the chest with each inspiration.

At 3½ P. M. on the third, I performed tracheotomy, as death seemed imminent without it. Immediately the breathing was relieved.

We gave him whisky freely, and gr. ii sulph. quinæ, in solut.on, every two hours.

4th. Feels pretty well. When the mucus is well cleared out of the tube, he breathes very easily, and without any effort. There is no lividity of face; pulse 140; of good strength; ate an egg.

*Vespere.* There was a good deal of mucus in the tube and below it, which was expelled with difficulty; continue quinine and whisky.

5th. I was called at 5½ A. M.; found him livid, and breathing with considerable effort, but after a time he brought up a greet deal of mucus, hardened into pellets, and very offensive in its character, This ejection of mucus was repeated at 12 M. and at 7 P. M. Stomach will retain nothing; pulse 120; very feeble; fauces now clear of membrane; sinapisms to the epigastrium, and injection of beef tea. egg and quinine.

6th. I was called to see him again at 1 A. M.; found him in great distress, with much difficulty of breathing; after a time he expectorated mucus and seemed relieved; he swallowed a good dose of quinine and more than a table-spoonful of whisky; these he retained and fell asleep breathing easily. In the morning he became worse, and died at 8 A. M., surviving the operation 64 hours. There was no post mortem examination.

Dr. J. E. Clark and myself saw this patient, alternately, every three or four hours from the time of the operation to his death.

Both of these last cases, although unsuccessful, are corroborative of the propriety of tracheotomy in diphtheria, when the larynx is affected and death imminent therefrom.

In both, life was prolonged more than two days, and an opportunity for the "vis medica rix naturæ," aided by powerful tonic and nervine remedies, to restore the interrupted functionc of the system.

It is an operation in which everything is to be gained, *and nothing can be lost.* If one patient out of seven or eight is saved, it is a full reward for all the trouble and anxiety consequent on these sad cases.

REMARKS BY DR. JAMES M. MINOR.

Dr. Minor thought Dr. Gilfillan's case one of much interest, and of the first importance, inasmuch as it established the fact—as far as one case could—that tracheotomy could be safely resorted to in diphtheritic croup. Dr. M. had also had some years since a successful case of tracheotomy. He thought the general disfavor in which tracheotomy was held by the profession, more especially in diphtheria, was not based upon sound principles. If it was apparent that the patient must die, and that by gradual suffocation, the most agonizing and prolonged of all deaths, why not give this additional chance for life, even though it were smaller than it really is? The depressing effects of the operation were not to be compared to even ten minutes of the dreadful struggles for breath of the helpless little sufferers. For if the patient dies soon after the operation, death is then comparatively easy. The surgeon has the satisfaction of knowing that he has given some hours, perhaps days, of relief from the most terrible agony, and the parents and friends have the consolation of knowing that the path to the grave of a dear little one has been cleared of its worst horrors.

Dr. Minor thought that there were in the contemplation of this operation two distinct points requiring special consideration. These were, *when to operate* and *the treatment after operation.* The operation itself, although difficult, and requiring great care, was of secondary importance, compared with the time when it should be performed and the subsequent management. If after the failure of all ordinary means of relief the patient begins to manifest symptoms of exhaustion and has discoloration of the prolabia, the operation should not be delayed a moment, for at such times moments are precious—they decide the fate of the patient. It may be true that cases have recovered without operation which had been considered hopeless, and indeed, a most remarkable coincidence illustrating this had occurred under his own observation. Two children, living opposite each other on the same street, were similarly affected, and under treatment at the same time. In one tracheotomy was resorted to, and in the other, under the care of Dr. H. S. Smith, general treatment was relied upon. Both cases recovered. Such illustrations, however, were rare, and are not to be taken as criteria.

Success, Dr. M. thought depended more upon after-treatment of tracheotomy than in any and every other operation in surgery. Untiring assiduity and sleepless vigilance were imperatively demanded, without which few cases will succeed; with this care we may reasonably hope for a fair proportion of success.

Dr. M. uses Trousseau's double canula as modified by himself, having a fenestrum in the inner canula corresponding to the one in the outer, and, at a point corresponding as near as may be with the larynx,' the inner canula projecting slightly beyond the outer. and the general curve, such as that when in positión, the lower portion shall correspond with the axis of the trachea. Dr. Minor considers the inner fenestrum of much importance as it allows frequent tentative experiments upon the permeability of the larynx to air by the insertion of a velvet cork in the external opening of the canula, by which means the earliest possible information is gained as to the proper time for the removal of the canula, a point of much importance both as regards the removal of so large a foreign body as a canula from so delicate a region, and the earliest possible restoration of the larynx to functional activity. The projection of the inner canula serves to push off the inspissated mucus which obstructs the lower end of the outer canula, and which would otherwise be a serious obstruction to respiration. The internal canula should be removed frequently, cleansed repeatedly, and greased inside and out with *lard*, (not oil;) the outer canula should have short flanges to be held in place by India-rubber tapes round the neck, which allow the canula free play, and are not liable to stiffen with the secretions, An intelligent and thoroughly instructed nurse should always be in attendance, never leaving the patient. The surgeon should see the child every two or three hours if possible, and be at all times within call. A warm, moist atmosphere is very important.

